# Characterization of the Metal Fused Filament Fabrication Process for Manufacturing of Pure Copper Inductors

**DOI:** 10.3390/ma16206678

**Published:** 2023-10-13

**Authors:** Philipp Schüßler, Jonas Franke, Steffen Czink, Steffen Antusch, Daniel Mayer, Stephan Laube, Thomas Hanemann, Volker Schulze, Stefan Dietrich

**Affiliations:** 1 Institute for Applied Materials—Materials Science and Engineering (IAM-WK), Karlsruhe Institute of Technology (KIT), 76131 Karlsruhe, Germany; 2 wbk Institute of Production Science, Karlsruhe Institute of Technology (KIT), 76131 Karlsruhe, Germany

**Keywords:** fused filament fabrication, material extrusion, metal, copper, microcomputed tomography, porosity, electrical conductivity

## Abstract

This work presents a comprehensive investigation into the optimization of critical process parameters associated with metal fused filament fabrication (Metal-FFF) for the production of copper-based components. The study focused on three different commercial and one self-manufactured filament, each with unique chemical compositions. These filaments were systematically optimized and the density was characterized for all processing steps, as well as the electrical conductivity on the specimen scale. Remarkably, two of the studied filaments exhibited exceptional properties after sintering with forming gas (up to 94% density and 55.75 MS/m electrical conductivity), approaching the properties measured for established manufacturing methods like metal injection molding. Finally, the research was extended to component-scale applications, demonstrating the successful fabrication of inductors with integrated cooling channels. These components exhibited water tightness and were used in induction hardening experiments, validating the practical utility of the optimized Metal-FFF process. In summary, the results show great promise in advancing the utilization of Metal-FFF in industrial contexts, particularly in the production of high-performance copper components.

## 1. Introduction

In contrast to subtracting and forming manufacturing methods, additive manufacturing (AM), often referred to as 3D printing, produces components from 3D model data by joining materials, usually layer by layer [1]. AM has gained enormous attention in recent years, but transitioning from research to industrial applications is challenging due to its relatively high cost per unit and slow speeds [2,3] compared to conventional processes such as forming [4]. Therefore, AM is often used in markets where only small quantities of highly optimized parts are needed, such as the medical, aerospace and space industries [2,3]. AM enables one to produce optimized geometric features and allows the integration of multiple functions or even sensors directly into parts that are challenging to manufacture or integrate by conventional techniques. While the fused filament fabrication technique (FFF; often referred to as extrusion-based additive manufacturing, MEX) is widely used for polymers or composites [5,6,7,8,9], metal parts are generally manufactured by powder bed fusion processes (e.g., selective laser melting (PBF-LB), electron beam melting (PBF-EB)) or indirect multi-step processes combined with sintering (e.g., binder jetting (BJT)) [10,11,12,13,14,15,16]. The current AM processes for metals produce parts with a relatively high bulk density (greater than 99% for PBF-LB or PBF-EB) but the drawbacks are high machine and operation costs as well as the safety risk of handling the fine metal powders. Since these processes are based on melting the metal during the process by a laser or electron beam, metals like pure copper are challenging to process due to the high thermal conductivity. Metal-FFF processes can produce high-quality parts on affordable off-the-shelf 3D printers normally intended for use with polymers like PLA or ABS, with virtually no changes required. For the Metal-FFF process, one or more polymeric binders are used in combination with fine metal powder particles to create the feedstock for the filaments. The feedstock for Metal-FFF is comparable to the feedstock used for metal injection molding (MIM). Recent studies have shown that it is possible to use the same feedstock for both processes [17,18]. For usage with a commercial FFF 3D printer, a filament with a typical diameter of 1.75 mm or 2.85 mm is created through an extrusion process. The polymeric binders are used as a backbone during 3D printing and are chemically and/or thermally removed after the printing process, in a step called debinding. The debound part is then sintered (heated to a temperature slightly below the melting point of the metal) in an oven to create the final part. The Metal-FFF process was successfully used to create parts out of different steels [19,20,21], titanium alloys [22,23,24] or pure copper [25,26,27,28]. Most research for Metal-AM is centered around the manufacturing of an optimized binder system [24,29,30] or the usage of closed-off proprietary ecosystems (e.g., Metal-X system [25,27]) to study the mechanical properties and the density of the sintered parts [25,27]. While these filaments are more affordable than other methods of additive manufacturing, the cost will be even lower for off-the-shelf commercial filaments usable with consumer-grade FFF 3D printers.

This work aims to analyze three commercial filaments filled with pure copper powder with a self-manufactured filament on consumer-grade FFF 3D printers to create a usable demonstrator on the component scale. A new filament based on a kerosene wax, LDPE and stearic acid as a binder was produced and analyzed. A parameter study with the four filaments was conducted for all steps of the process chain from 3D printing to the sintered parts. The parameter study included printing parameters (e.g., printing speed and temperature, extrusion multiplier), solvent-based debinding (solvent temperature and time) and the thermal-based debinding and sintering (sintering atmosphere). A usable demonstrator on the component scale (copper inductor with internal cooling channel) was manufactured, analyzed and tested.

## 2. Material and Methods

### 2.1. Materials

Three commercially available (1.75 mm diameter) and one self-manufactured copper filament (2.85 mm diameter) were used for this study. All four filaments can be used with consumer-grade FFF 3D-printers. The details of the used filaments are listed below and in Table 1.

- Filament F1: Commercial copper filament (Copper Filamet, The Virtual Foundry Inc., Stoughton, WI, USA) containing about 89 wt.% copper and one unspecified polymer as a binder.
- Filament F2: Commercial copper filament (Cu, PT+A GmbH, Dresden, Germany) containing about 93 wt.% copper and two unspecified polymers as binders.
- Filament F3: Commercial copper filament (AM-X Cu Excellence, AM Extrusion GmbH, Radebeul, Germany) containing more than 93 wt.% copper and two unspecified polymers as binders.
- Filament F4: Self-manufactured copper filament containing 93 wt.% copper with a D50 of 1.65 μm (CU-110, Atlantic Equipment Engineers Inc., Upper Saddle River, NJ, USA) and two polymers as binders. The binder system used for the manufacturing of the custom filament consisted of kerosene wax, namely Sasolwax 6403 (Sasol Ltd., Johannesburg, South Africa), and LDPE (Lupolen 1800H, LyondellBasell Industries N.V., Rotterdam, The Netherlands). A dispersant, stearic acid (Carl Roth GmbH + Co. KG, Karlsruhe, Germany), was added to the binder system as an additive. The non-polar kerosene wax served as a primary binder and non-polar polyethylene as a secondary binder.

### 2.2. Filament F4 Feedstock Compounding

The feedstock for the self-manufactured filament F4 was prepared in a mixer–kneader compounder (W50-EHT, Brabender GmbH, Duisburg, Germany) with a rotation speed of 30 rpm, a compounding temperature of 125 °C and a processing time of 1 h. The produced feedstock was then extruded into filaments with a diameter of 2.85 mm using a Noztek single-screw extruder (Noztek pro, Noztek, UK). Further, the temperature- and shear-rate-dependent melt flow was characterized by a high-pressure capillary rheometer (Rheograph 25, Goettfert Werkstoff-Pruefmaschinen GmbH, Buchen, Germany).

### 2.3. Metal Fused Filament Fabrication (Metal-FFF)

**Figure 1:**
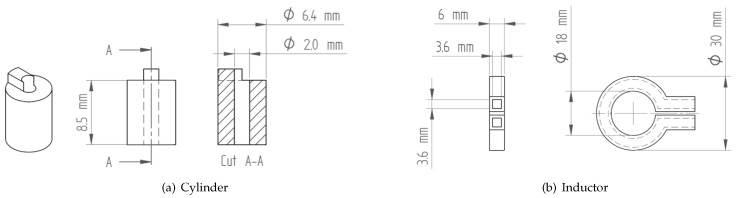
Geometric dimensions of the (**a**) cylindrical specimen and (**b**) inductor for the component-scale test.

The 3D printing of the parts was performed on a Prusa i3 MK3S+ (Prusa Research a.s., Praha, Czech Republic) for the three commercial filaments with a diameter of 1.75 mm and on a German Rep Rap X350pro (innovatiQ GmbH & Co. KG, Düsseldorf, Germany) for the self-manufactured filament with a diameter of 2.85 mm. A 0.4 mm diameter hardened steel nozzle was used for filament F3, while 0.6 mm diameter hardened steel nozzles were used for the other filaments. In order to avoid cross-contamination, a separate nozzle was used for each of the four filaments. Two different geometries were created for this work. In order to optimize the process parameters, a simple hollow cylinder was used (Figure 1a). An added feature on the top of the hollow cylinder allowed for the alignment of the sample during microcomputed tomography (μCT) analysis in order to trace specific pores through each process step. To validate the results of the process optimization on the specimen scale, an inductor with an integrated cooling channel was selected as a demonstrator on the component scale (Figure 1b). This inductor was printed flat on the buildplate without a support structure, as well as inclined to 45 deg with a support structure beneath the inductor.

### 2.4. Debinding and Sintering

**Figure 2:**
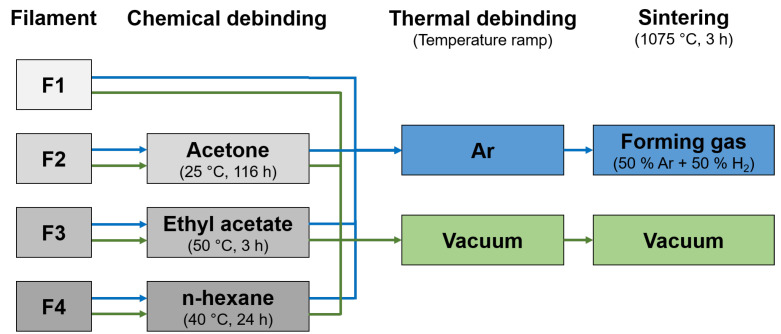
Visualization of the used processing routes for filaments F1–F4. The forming gas route is highlighted in blue and the vacuum route in green.

Generally, the debinding process is divided into two separate steps (solvent- and thermal-based debinding) to remove all binders from the 3D-printed part (green part). Since filament F1 only used a single polymer as the binder, the process of solvent-based debinding could not be used for this filament. For the solvent-based debinding step, acetone was used for filament F2 and ethyl acetate for filament F3, as well as n-hexane for filament F4 [24], in order to remove the low-molecular-weight part of the primary binder. The debinding with ethyl acetate was investigated for different temperatures and debinding times on a ROTILABO MH 15 heating and magnetic stirrer (Carl Roth GmbH & Co. KG, Germany). The debinding with n-hexane was performed in a homemade glass reactor extraction apparatus equipped with a reflux condenser [24]. In order to determine the efficient debinding time, samples were removed after specific time intervals and dried for 60 s under a hot air blower and for 24 h under a cold air blower. They were then weighed and the weight loss due to the solvent-based debinding was calculated. To validate the drying, some samples were dried in a vacuum drying oven (Memmert GmbH & Co. KG, Schwabach, Germany) for two days and the weight was compared with the weight before drying in the vacuum oven. The thermal-based debinding and sintering steps were performed in a two-step process. Two different sintering atmospheres were investigated and used for all four filaments in this study (see Figure 2). For the reference process (Figure 2, blue), an Ar atmosphere was used for thermal debinding (Carbolite GLO 10/11-2G, Carbolite Gero GmbH & Co. KG, Neuhausen, Germany) and a forming gas atmosphere (50 vol.% Ar, 50 vol.% H${}_{2}$) was used for the sintering process (Carbolite HTK 8Mo/14-1G, Carbolite Gero GmbH & Co. KG, Germany). A vacuum oven (vacuum pressure of 25 mbar) was used for comparison (ALD Vaccum Technologies GmbH, Hanau, Germany). The temperature profile during the thermally based debinding step was optimized for all four filaments by thermogravimetric analysis. An argon atmosphere was used for the thermogravimetric analysis with a heating rate of 1 K s^−1^ to a maximum temperature of 600 °C on an STA 449 F1 Jupiter (Netzsch Gerätebau GmbH, Selb, Germany). For a better comparison, the same temperature profile was used for the thermally based debinding and sintering, independent of the used filament type.

### 2.5. Characterization

All specimen-level density measurements were conducted on a precision scale (Mettler Toledo, Columbus, OH, USA). The weight of the samples was first measured in air and then in water. The density ρ was calculated by the Archimedes method (Equation (1)), with the measured weight in air $w_{air}$, the measured weight in water $w_{water}$ and the temperature-dependent density of water $\rho_{water}$. A dispersive agent was used to reduce the surface tension.

(1) $$\rho =\frac{w_{air}\;\cdot \;\rho_{water}}{w_{air}-w_{water}}$$

For a detailed investigation of the distribution and geometry of the pores, five samples of each filament and processing step were investigated with μCT. μCT images were recorded on a YXLON Precision μCT (YXLON International GmbH, Hamburg, Germany) using an acceleration voltage of 180 kV and a target current of 0.1 mA. For each reconstruction, 2070 projections over a 360 deg rotation were recorded on a Perkin Elmer XRD1620 AN flat panel detector with 2048 pixel × 2048 pixel with a pixel pitch of 0.2 mm. Samples were scanned at a focus object distance (FOD) of 18.5 mm and a focus detector distance (FDD) of 900 mm, resulting in a voxel size of 4.1 μm. A detector integration time of 1 s and frame binning with two frames were selected to reduce noise in the projection images. Image reconstruction was carried out using the filtered backprojection (FBP) algorithm in VGStudioMAX 2022.4 (Volume Graphics International GmbH, Heidelberg, Germany). For further noise reduction, a median filter was applied as well. The porosity was analyzed with the VGEasyPore module implemented in VGStudioMAX.

The electrical conductivity of the sintered copper specimens was measured using the eddy current instrument SIGMATEST 2.069 (Institut Dr. Foerster GmbH & Co. KG, Reutlingen, Germany). The measured electrical conductivity was compared to annealed pure copper with respect to the International Annealed Copper Standard (IACS), where the reference value of 100% IACS is $58\times 10^{6}$ S m^−1^ [31]).

## 3. Results and Discussion

### 3.1. Process Parameter Optimization

**Figure 3:**
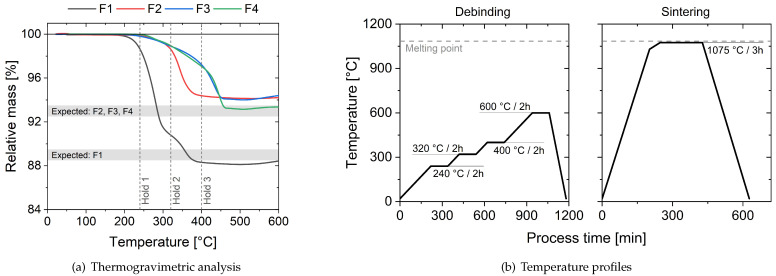
Visualization of the (**a**) thermogravimetric analysis for filaments F1–F4. The selected holding steps for the thermal debinding process are highlighted with short dashed lines and the claimed relative copper mass fractions with a light grey background. The starting weight was selected as a reference for the relative mass. Also shown is the (**b**) temperature profile used during the thermal debinding and sintering process for both processing routes.

A holistic study for the optimization of the most important FFF process parameters was conducted for this work. The printing parameters were first selected according to the manufacturer suggestions and then systematically optimized for the used geometry and FFF printers. As the printing temperature is strongly dependent on the chemical composition of the filament (used polymeric binders), it was tailored to each specific filament. The printing temperature is reported to directly affect the print quality, leading to the high viscosity of the extruded filament at low printing temperatures (clogging of the nozzle [29]), to a low viscosity (dimensionally unstable, over extrusion [29]) or to the gaseous decomposition of the polymeric binders at too high printing temperatures (see Figure 3). After optimization, the printing temperatures were set to 215 °C for filament F1, to 145 °C for F2, to 130 °C for F3 and to 140 °C for F4 (See Table 2). Since the scope of this study was limited to the maximization of the density and not the highest build rate, relatively slow base printing speeds of 40 mm s^−1^ were used as a starting point in this study. A lower printing speed is reported to reduce process-induced porosity between adjacent deposited strands [32]. Thus, the printing speed was incrementally reduced for every filament until no further increase in density was noted. This resulted in the optimized printing speeds shown in Table 2, which differed for the used filaments. The extrusion multiplier (i.e., flow rate) controls the mass flow through the nozzle and therefore significantly influences the density of the printed specimens. A higher extrusion multiplier reduces process-induced porosity between adjacent deposited strands as it forces more material into these voids. If the extrusion multiplier is set too high, the overflow of material results in low dimensional accuracy or the complete failure of a print due to a collision with the nozzle [29]. A concentric infill strategy with 100% infill was used for this study. After optimization, the extrusion multipliers were set to 1.2 for filament F1, to 1.3 for F2, to 1.2 for F3 and to 1.05 for F4.

For this study, the debinding process was split into two separate steps. The solvent-based debinding step was used to remove the low-molecular-weight part of the primary binder. Since filament F1 only used a single polymer as the binder, the process of solvent debinding was omitted for this filament. As the effectiveness of solvents is strongly dependent on the used low-molecular-weight polymer, different solvents were used for each filament. Acetone at room temperature was used for the solvent-based debinding of filament F2, ethyl acetate at 45 °C to 55 °C was used for F3 and n-hexane at 40 °C was used for F4. To achieve the desired debinding results (according to manufacturer data for F2 and F3 or the mixture ratio for the self-manufactured filament F4), the debinding time was varied until no further mass loss was measured (Table 3). This resulted in a debinding time of 116 h for F2, 3 h for F3 and 24 h for F4.

After the solvent-based debinding step, the thermal-based debinding and the sintering step were performed in a two-step process. Since the thermogravimetric analysis showed a significant difference in the decomposition rates and temperatures between the four filaments, multiple holding steps were programmed. The mass loss over temperature as well as the selected holding temperatures are visualized in Figure 3. The resulting relative mass after chemical and thermal debinding was consistent with each manufacturer’s claimed relative copper mass fraction. The holding steps were designed to give enough time for the formed gases to escape from the inside of the specimens through the pores created by the solvent-based debinding step. The sintering temperature and time were varied in order to optimize the resulting density of the specimens. After optimization, it was set to 1075 °C, 10 °C below the melting point of pure copper, for 3 h. The temperature profile is shown in Figure 3b (left) for the thermal-based debinding and in Figure 3b (right) for sintering.

### 3.2. Metal-FFF on Specimen Scale

**Figure 4:**
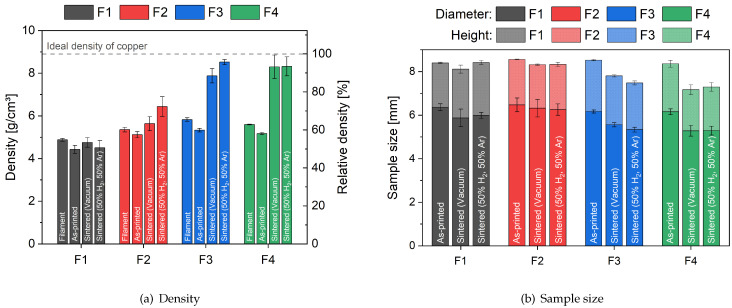
Visualization of (**a**) measured density and relative density of as-printed, chemically debound, vacuum-sintered and forming-gas-sintered specimens and (**b**) measured diameter and height of as-printed, vacuum-sintered and forming-gas-sintered specimens.

Density measurements according to the Archimedes method and reconstructed μCT scans were carried out on all hollow cylinder specimens to elaborate the global density after each manufacturing step (Figure 4). The optimized parameters for the Metal-FFF 3D printing process, shown in Section 3.1, were used for the manufacturing of these specimens. With a density of 4.88 $g\;cm^{-3}$, filament F1 showed the lowest filament density. The densities of filaments F2 and F4 were calculated as 5.36 $g\;cm^{-3}$ and 5.59 $g\;cm^{-3}$, respectively, and the highest filament density was measured for F3 with 5.83 $g\;cm^{-3}$. All filaments show a slightly lower density in the as-printed state compared to the original filament before printing. The highest density of the printed parts was also noted for filament F3. Due to the open porosity after the chemical-based debinding process, no density measurements were possible with the Archimedes method for this step.

After thermal debinding and sintering in the vacuum oven, filaments F3 and F4 showed the highest density with 7.88 $g\;cm^{-3}$ and 8.30 $g\;cm^{-3}$. When compared to conventionally manufactured pure copper (8.9 $g\;cm^{-3}$), this corresponds to 88.5% or 93.3%, respectively. While these two filaments reached a density comparable to state-of-the-art processes like metal injection molding (MIM) (about 95% density [33]) or selective laser melting (about 95% to 98% density [34,35]), filaments F1 and F2 showed a significantly lower density after vacuum sintering (53.5% for F1 and 63.4% for F2). Only two filaments showed a significant improvement in the resulting density while using Ar during the thermal debinding and the forming gas mixture during sintering. Filament F2 reached a density of 83.6% and filament F3 a density of 95.7%, the overall highest density of this study. However, no improvements in density were noted for filaments F1 and F4. The density was further supported by the measured sample diameter and height. Consequently, samples with high density showed greater shrinkage during sintering (Figure 4b).

**Figure 5:**
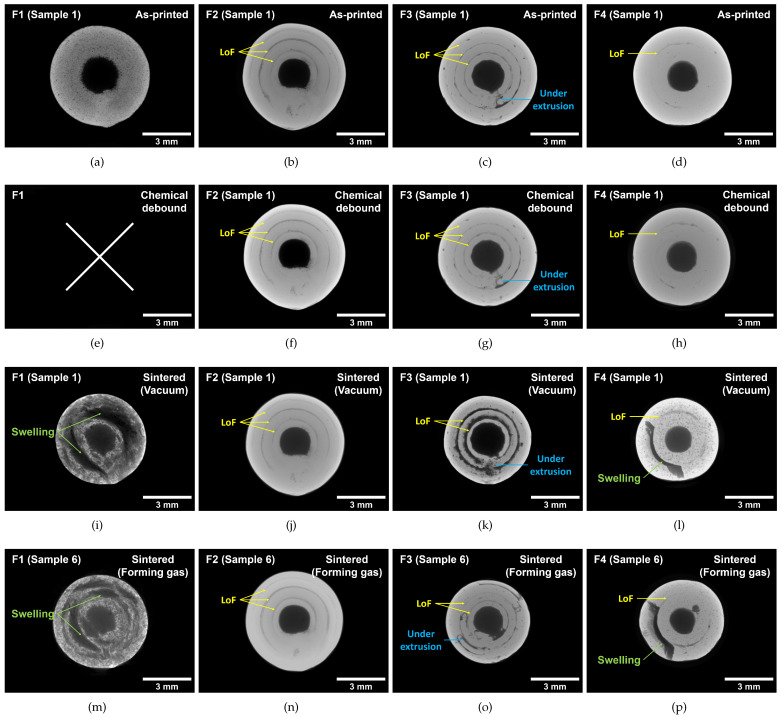
Minimal grey value projections with a height of 4mm, reconstructed from μCT scans for (**a**–**d**) the as-printed specimens S1, (**e**–**h**) the same specimens after chemical debinding, (**i**–**l**) specimens S1 after sintering in vacuum and (**m**–**p**) specimens S6 after sintering in forming gas atmosphere for filaments F1–F4. Lack of fusion (LoF) defects are highlighted in yellow, under-extrusion in blue and swelling and cracks induced by trapped gases in green.

To further elaborate on the cause of the porosity, μCT analysis was performed on the 3D-printed, chemically debound and sintered specimens. Five specimens of each filament were manufactured and analyzed for each process route (Figure 2). Only one sample of each filament and process route (sample 1 for the vacuum route and sample 6 for the forming gas route) is shown in this section, but these are representative of all analyzed specimens. With the use of this non-destructive analysis at every step of the process for the same specimens, specific pores can be traced back to the exact cause. This allows us to distinguish between manufacturing-induced porosity during 3D printing and residual porosity induced by the debinding and sintering process. Figure 5 shows the reconstructed μCT projection images for (a–d) the as-printed, (e–h) chemically debound, (i–l) vacuum-sintered and (m–p) forming-gas-sintered cylinders. All shown μCT images are projections of 4 mm depth, showing the minimum grey value. Most of the defects visible after sintering can be directly traced back to the as-built specimens. These defects are known as lack of fusion (LoF) or under-extrusion effects, mostly located between adjacent printed lines of the specimen (see Figure 5). Minimal LoF defects were noted for filament F4, while a severe lack of fusion defects was noted for filaments F2 and F3. Consequently, the printing parameters for these three filaments could be improved further. Further defects such as cracking and swelling can be traced back to the thermal debinding and sintering process. The cracking and swelling was most prominent for the specimens of filament F1, resulting in a low-density part. The swelling and cracking can be attributed to the single binder system, resulting in trapped gases during the thermal debinding process. Localized swelling and cracking was also noted for the specimens of filament F4. These defects followed the LoF pores between to adjacent printed lines and could also be the result trapped gases during the thermal debinding process. No swelling effects were noted for the other filaments F2 and F3.

The combination of low global porosity, low shrinkage and almost no visible LoF pores led to the assumption that the specimens printed with filament F2 contained many small pores not visible in the μCT reconstruction. Only minimal differences were noted between samples sintered in the vacuum oven or with forming gas. The forming gas atmosphere helped to reduce the porosity for filament F2, but the relative density was significantly lower compared to filaments F3 and F4.

**Figure 6:**
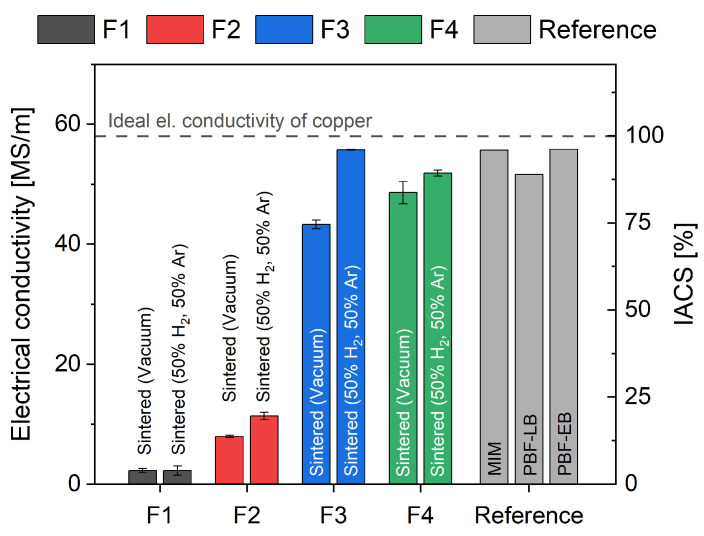
Measured electrical conductivity of the specimens after sintering in vacuum and in forming gas atmosphere for filaments F1–F4. The MIM process [33], the PBF-LB process [35,36] and the PBF-EB process [12,13] were used as reference.

In addition to the density, the electrical conductivity is of significant importance for most applications of pure copper parts. The measured electrical conductivity of the vacuum-sintered and forming-gas-sintered specimens is highlighted in Figure 6, complete with a comparison to the IACS reference and other manufacturing methods, such as metal injection molding (MIM) and laser-based (PBF-LB) and electron-beam-based powder bed fusion processes (PBF-EB) [12,13,33,35,36]. With 84% of the IACS, specimens manufactured with filament F4 reached the highest electrical conductivity, followed closely by filament F3 with 75% of the IACS. The noted electrical conductivity for filaments F1 and F2 was significantly lower, with only 2% to 20% of the IACS. Other metal-based additive manufacturing methods will reach similar electrical conductivity as for filaments F3 and F4 [12,13,33,35,36]. The electrical conductivity can be directly correlated to the effective cross-sections of the specimens, and therefore to the density shown in Figure 4. The forming gas sintering was able to improve the electrical conductivity of all filaments, with significantly better results for filament F3, reaching the highest electrical conductivity of 55.75 MS/m (96% of the IACS). The improvements in both density and electrical conductivity can be explained by the reduction of copper oxides by the hydrogen used in the forming gas mixture [37].

### 3.3. Metal-FFF on Component Scale

Filament F3 was chosen to print the specially designed inductors with integrated cooling channels for the component-scale analysis. The filament was selected due to the combination of high density (95.7%), high electrical conductivity (96% of the IACS) and the good printablity. Samples were printed with different orientations and support structures, as further described in Section 2.3. The process route of chemical debinding, thermal debinding with Ar and sintering with the forming gas was selected, due to the higher resulting density and electrical conductivity. The measured shrinkage of 14–16% was in line with the data from the specimen-scale experiments.

**Figure 7:**
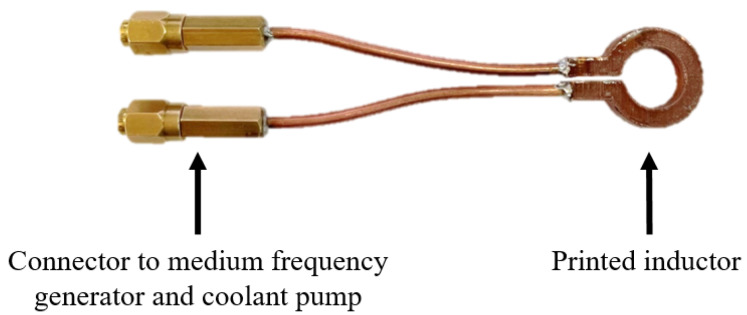
Inductor manufactured with the Metal-FFF process chain, finished with connectors to the medium-frequency generator and coolant pump.

In order to test the inductors, they were brazed to small copper pipes and fittings (Figure 7). This additional hardware was needed as a connector to the medium-frequency generator as well as the coolant pump. The inductors were successfully tested regarding water tightness of up to 800 kPa of coolant pressure for an hour. Furthermore, the inductors were then used for multiple induction hardening experiments. For these experiments, bars of C45 steel (1.0503) were successfully heated above the austenitization temperature in under 10 s. The electrical conductivity of the inductors was measured before and after the induction hardening experiments. Electrical conductivity comparable to the smaller specimen scale was characterized (94% of the IACS), with no changes noted after the induction hardening experiments.

## 4. Conclusions

In conclusion, this work presents a comprehensive study of the optimization of the fused filament fabrication (FFF) process parameters for the production of copper-based components using Metal-FFF technology. The key findings and results of this study shed light on the critical factors influencing the quality and properties of the printed parts.

- Filament-specific optimization: The study highlights the importance of tailoring the printing parameters, such as the nozzle temperature and extrusion multiplier, to the specific chemical composition of each filament. This approach is critical to achieving optimal print quality and avoiding problems such as clogging or over-extrusion.
- Density: The results indicate that two filaments, F3 and F4, show the highest density after sintering, achieving densities close to those with conventional manufacturing methods such as metal injection molding (MIM). The use of Ar during thermal debinding and a forming gas mixture during sintering was found to be effective in improving density. Meanwhile, the μCT analysis revealed that printing defects such as a lack of fusion pores could not be closed during sintering.
- Electrical conductivity: The study also examined electrical conductivity, a critical property for copper-based components. Filaments F3 and F4 exhibited superior electrical conductivity after forming gas sintering, making them suitable for various applications requiring high conductivity.
- Component-scale application: The research was extended to component-scale applications, demonstrating the practicality of the optimized Metal-FFF process by successfully printing inductors with integrated cooling channels. These components were tested for water tightness and successfully used in induction hardening experiments.

Overall, this study provides valuable insights into the Metal-FFF process for copper-based components, offering a pathway to enhance both the density and electrical conductivity. The optimized parameters and process steps outlined in this research hold great promise in advancing the use of the Metal-FFF technology in various industrial applications, particularly in the production of high-performance copper components.

## Figures and Tables

**Table 1:** Differences in the four used filaments, F1–F4.

Filament	Diameter	Copper Content	Copper Content	Manufacturer
F1	1.75 m m	89 wt.%	46 vol.%	The Virtual Foundry Inc.
F2	1.75 m m	93 wt.%	56 vol.%	PT+A GmbH
F3	1.75 m m	93 wt.%	61 vol.%	AM Extrusion GmbH
F4	2.85 m m	93 wt.%	60 vol.%	Self-manufactured

**Table 2:** Optimized process parameters for the manufacturing process with filaments F1–F4.

Filament	Filament Diameter [mm]	Nozzle Diameter [mm]	Nozzle Temperature [°C]	Buildplate Temperature [°C]	Layer Height [mm]	Extrusion Multiplier [-]	Extrusion Speed [mm/s]
F1	1.75	0.6	215	60	0.05	1.2	20
F2	1.75	0.6	145	60	0.2	1.3	30
F3	1.72	0.4	130	65	0.15	1.2	30
F4	2.85	0.6	140	60	0.1	1.05	10

**Table 3:** Recorded mass loss during chemical debinding for filaments F1–F4.

Filament	Solvent	Mass Loss/%	
		**Target**	**Measured**
F1	No solvent	-	-
F2	Acetone (25 °C for 116 h)	4.1–4.7	4.28±0.12
F3	Ethyl acetate (50 °C for 3 h)	3.5–4.5	3.53±0.03
F4	n-hexane (40 °C for 24 h)	3.0	2.96±0.02

## Data Availability

The data presented in this study are available on request from the corresponding author.
